# Contaminated area instability along Ångermanälven River, northern Sweden

**DOI:** 10.1007/s10661-017-5839-0

**Published:** 2017-02-20

**Authors:** A. Ströberg, K. Ebert, J. Jarsjö, A. Frampton

**Affiliations:** Department of Physical Geography, Stockholm University, S-10691 Stockholm, Sweden

**Keywords:** Local soil contamination, Slope instability, Rivers, Mass movement, Risk assessment

## Abstract

Industrially utilized river basins are frequently exposed to contaminants originating from polluting activities. However, the physical instability and probability of mass movement mobilization of contaminated soil into rivers have only received little attention. In this study, we present a GIS-based method to produce a regional overview of where and how contaminated areas are potentially exposed to slope instability. A landslide susceptibility-index was used to study the degree and distribution of overlap between contaminated sites and unstable ground. A contaminated area instability hazard classification was produced integrating slope instability and contamination risk classification. Our results indicate that mass movement can be tied mainly to a slope gradient ≥16°, a proximity to the river that is <500 m, a distance of <500 m from roads, concave surface curvature, and sand- and silt soils. Forty-six (22%) of all considered contaminated sites are located within areas with a non-negligible slope instability, of which a majority, 30 sites (14%) are situated on ground with a low or moderate instability. Three sites with a class 2 contamination risk (the 2nd highest class) are located on ground with a very high slope instability.

## Introduction

It is today generally acknowledged that the result of a risk-assessment is usually altered when combining natural and human-induced hazards (Holden and Jacobson 2013; Kappes et al. 2012). However, the methodological approach as to what constitutes a multi-hazard has resulted in a general failure to recognize that, although some hazards may pose limited environmental or health risks in isolation, they can interact to considerably increase risks if they overlap geographically. For instance, no thorough framework is in use today for investigating slope instability and contaminated soil as a combined hazard, even though they frequently are overlapping in the same system.

Slope processes such as landslides are natural occurrences and usually not considered a risk if they do not affect humans or infrastructure directly. Slope processes can however pose an indirect risk by spreading contaminants, especially when contaminated soil is sliding into rivers. The concept of slope processes as a pathway for contaminants has only been given recognition in recent years, mainly through the work of Göransson et al. (2009, 2012, 2014). Sediments deposited in streams and rivers from mass movement have a natural impact on water quality (Göransson et al. 2012) and channel morphology (Brydsten et al. 1990; Korup 2004; Schuerch et al. 2006; Svensson et al. 2006; Inoue et al. 2012). The impact of hazardous contaminants bound to these sediments is a human-induced risk through which the consequences may vary based on a number of different factors, e.g., type of contaminants, soil types, water properties, and types of slope process (Schoor 1996; Yong and Mulligan 2003). Organic and non-organic hazardous substances from these activities have a varying potential of binding to soil (Huang et al. 1977; SEPA 2009), from which they can spread into a river through slope processes and river bank erosion.

Current research approaches multiple-hazards through adding up all relevant hazards within an area, or as one process triggering another in a domino- or cascade effect (e.g., Thierry et al. 2008; Carpignano et al. 2009; Marzocchi et al. 2009; MATRIX 2010; Schmidt et al. 2011; Bell and Glade 2012; DFID 2012; Kappes et al. 2012; Kreibich et al. 2014). Through a broadened perspective, based on multi-hazard analysis as discussed by Göransson et al. (2009) and Holden and Jacobson (2013) it is only when slope instability and local soil contamination are combined that the hazard becomes identifiable. Contaminated area instability might therefore be approached as a kind of synergic hazard, where two potential, and in many cases low-threat hazards in themselves, interact to create a more serious threat. The practical implications would be that contaminated area instability as an environmental hazard could otherwise be easily overlooked. This is especially true in areas where both processes have not been studied as independent hazards and the interdisciplinary connection might therefore not be made.

Connections between mass movements and the spread of contaminants have been mentioned briefly in reports from a small number of Swedish county administrative boards (Andersson 2013; Hultgren et al. 2014) as a potential future risk related to climate change. A national inventory of contaminated areas in Sweden has produced an estimate of around 80,000 contaminated or potentially contaminated areas in the country (SGU 2015). It is still largely unknown to what extent sediment-bound contaminants at these sites have been, and still are, exposed to mobilization due to mass movements. Even though the awareness of the hazard as a concept is evident at times, very little data has yet been produced to show the scale and distribution of contaminated area instability, leaving the scope of the problem an open question. By expanding on the information from Göta Älv River produced by Göransson et al. (2009, 2012, 2014) through looking at an additional location with different physical characteristics, a broadened understanding of the general proportions of and preconditions for the problem in Sweden can be obtained. Many rivers in northern Sweden share a similar geologic and industrial history to that of Ångermanälven River. A notable presence of contaminated area instability within the study area would therefore indicate that the problem could be more widespread in northern Sweden, although the frequency of mass movements is distinctively high along the Ångermanälven River (Christiansson and Arnér 1993; Svensson et al. 2006; SGI 2014). By analyzing underlying factors of slope instability along the river, the study will contribute to an understanding on how, when and where sediment-bound contaminants and mass movement might have interacted in the past, and where this type of hazard could become an issue in the future.

When sediments from mass movements release contaminants into streams the negative impact can be traced back to the industrial sites that are releasing the contaminants into the soil. This means that there are ways of preventing this process from taking place by identifying and preventing mass movements from occurring in areas with local soil contamination (e.g., intensive industrial activities and waste disposals), and by preventing the contamination of unstable grounds.

In areas where data on slope instability and local soil contamination is available, an overlay-analysis can illustrate where mitigation actions are most urgent. Methods for such an inventory, studies of the governing processes behind landslides spreading contaminants, and contamination transport from landslide-transported regolith into rivers have been suggested by Göransson et al. (2009, 2012, 2014). They used a regional spatial scale and provided useful information for risk reduction.

However where Göransson et al. (2009, 2012, 2014) was able to use already available data on slope instability along stretches of the Göta River, no sufficiently detailed data yet exists for Ångermanälven River. In regions where a detailed spatial inventory of slope instability is not available, an estimation of contaminated area instability is impossible. Since creating such a detailed inventory can be time consuming when covering larger areas, a quicker first-step estimation provides a helpful tool.

This study introduces an efficient method to gain a detailed, regional overview of where and to what extent contaminated areas are potentially being exposed to slope instability. The method is applied on Ångermanälven River, Sweden, exemplifying how spatial data on local soil contamination and basic landscape features can be combined in a GIS-application in cases where slope instability data is lacking, or where on site surveys are not possible. A resulting regional hazard inventory of the river and its surroundings will help in delineating, and prioritizing between areas of concern based on the combined hazard of overlapping local soil contamination and slope instability.

## Study area

**Fig 1:**
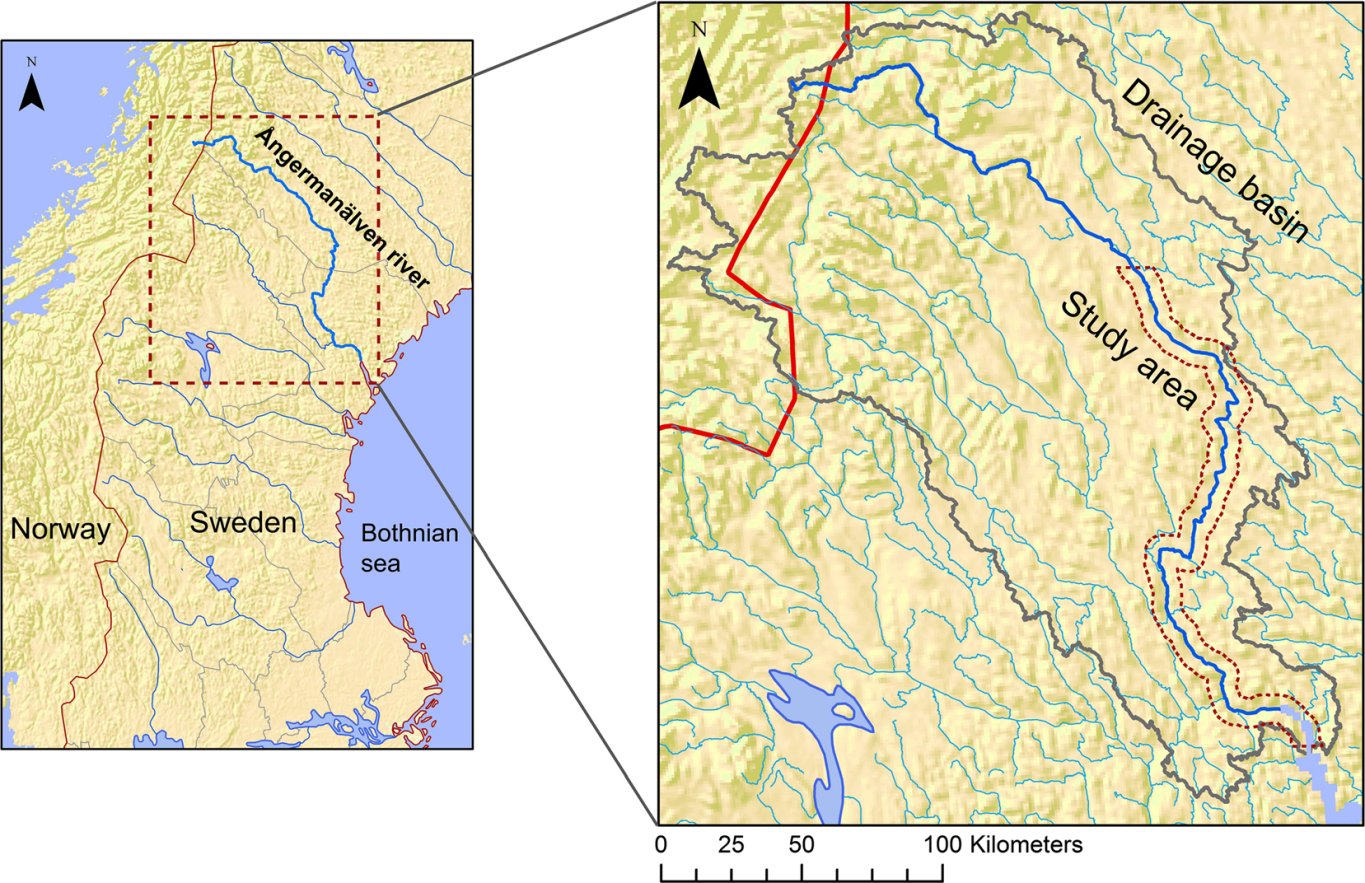
Location of the study area in Sweden

Ångermanälven River has its source in the Caledonian mountain range of northwestern Scandinavia. The river is 447 km long and drains in a southeasterly direction into the Bothnian Sea. The study area constitutes the southernmost 259 km of its length (Fig. 1).

The surroundings of the Ångermanälven River are in many places highly exposed to slope processes such as landslides, ravine-erosion, and undercutting of riverbanks from fluvial erosion, primarily because of isostatic land uplift (Christiansson and Arnér 1993; Berglund 2012). The region has a long industrial history based on primary and secondary industries such as lumber and metal processing plants, waste-dumps, and chemical factories among others. Most industries and infrastructure are located in close vicinity to the river banks because of the historically important use of running water for energy and as a means of waste disposal. Ångermanälven River has 15 hydroelectric power plants within the study area, some of which have adjoining water reservoirs. The soils are dominated by differing mixtures of sand and silt in the south and gradually coarser material such as gravel and cobble further north above the highest coastline. Because of the river’s high exposure to slope processes and industrial exploitation, research has been conducted to analyze landforms sensitive to slope processes (SGI 2014; Svensson et al. 2006) and to map local soil contamination (Länsstyrelsen Västernorrland 2011; Länsstyrelsen Västernorrland 2014a, b) along the river. To date, no study exists that integrates these factors along the Ångermanälven River.

Within the study area 209 contaminated or potentially contaminated areas have been identified and given a risk-grade on a scale of 1–4 where 1 marks the highest risk (SEPA 2002). The types of contaminants present in the study area are mainly determined by the kind of activities present at specific locations. A complete list of activities with the potential of spreading contaminants into their surroundings within the study area is available in Swedish at Västernorrland county administrative boards electronic GIS-database (Länsstyrelsen Västernorrland 2014b). The SEPA database (2002) identifies the most common sources of local soil contamination in the study area to be auto repair shops, sawmills with dipping, and waste disposals. The most high-risk sources of contaminants consists of processing industries such as concrete- and cement production, rubber production, iron and steel manufacturing plants, heavy metal foundries, waste disposals, and wood impregnation plants.

## Method

A landslide susceptibility index (LSI) is calculated, which results in spatial data showing five grades of slope instability. Above the slope instability map locations of local soil contamination are marked according to the four grade risk-scale set by SEPA, through guidelines determined in the MIFO-program (SEPA 2002, pp. 46). Through this map overlay, statistics and spatial data can be derived showing sites exposed to both potential slope processes and local soil contamination. By using a grading scale for both contamination risk and slope instability areas with a significant degree of exposure to both can be singled out for closer studies if deemed necessary. Overlay analysis has been used previously by Göransson et al. (2009) and proven to be a useful tool for producing spatial and quantitative data on contaminated area instability along the Göta Älv River. An LSI will expand the applicability of this method to regions where materials such as the landslide risk assessments, and in situ measurements used by Göransson et al. (2009) are unavailable.

### Slope stability mapping by landslide susceptibility-index method

The LSI-index method is a bivariate analysis used for estimating slope instability. The concept is based on identifying statistical correlations between previous mass movement events and physical landscape features on which these events have been triggered. The result is a slope instability-map and statistical data on the influence of physical landscape features on the preconditions for mass movements to occur within the study area. In contrast to what the name infers the method can also be used when incorporating susceptibility to other types of mass movement. The design of an LSI varies (Avinash and Ashamanjari 2010; Chalkias et al. 2014; Devkota et al. 2013; Jafaari et al. 2014; Shabanzadeh et al. 2011), but to the purpose of this study, i.e., to give a large-scale risk assessment based on limited data sources, the bivariate version which is more easily adapted to updated data was considered most suitable.

Initially a buffer zone is created stretching 5 km inland from the riverbanks of the Ångermanälven River. Within this area, sufficient spatial data exists on previous mass movement and landscape features that can be used for applying an LSI approach. Landscape parameters in raster format are added, based on previous studies of the preconditions and potential factors behind mass movement in the study area (Christiansson and Arnér 1993; SGI, Statens geotekniska institut 2014; Svensson et al. 2006), or areas of similar physical characteristics. Six parameters were used which are described below:

1. Slope gradient: One of the most common factors affecting slope instability in the study area is the slope gradient (Svensson et al. 2006). A slope-raster is created from a digital elevation model (DEM) (Lantmäteriet 2011) and divided into classes of 5° steps up to >45°. The layer resolution is converted from 2 × 2 to 20 × 20 m in order to decrease the likelihood that flat bottom-surfaces from scars and ravines are analyzed instead of the general slope gradient on which mass movements have been triggered.
2. Curvature: A curvature raster is created from the DEM (Lantmäteriet 2011) and allows for identification of crests and ravines that can be susceptible to mass movements and fluvial erosion. Curvature represents the convexity and concavity of every pixel within the study area. The layer is classified by an equal interval of 20, where a higher value indicates an upward and horizontally convex surface. A lower value indicates a concave surface.
3. Soil: The grain size of soil has a well-known influence on slope instability as it affects the cohesion and permeability of soils as well as the internal friction between grains (Beek et al. 2008). A digital soil-map (SGU 2014) is combined with a digitized analog map (Lundqvist 1987) in order for the whole study area to be covered. The map is converted into a raster with a 2 × 2 resolution.
4. Land Cover: Land cover can act to stabilize or to make slopes more susceptible to mass movement (Stokes et al. 2008). A CORINE land cover map (Lantmäteriet 2002) in raster format was used for this parameter.
5. *Distance to roads:* Vibrations from traffic and surface runoff from roads can act to make slopes more unstable, and act as a triggering mechanism for mass movements (Srbulov 2010). We use a multi-buffer with an interval of 100 m up to >500 m, originating from roads (derived from Lantmäteriet 2010) with a width of >5 mm, to include this parameter. Roads narrower than 5 m are considered to be too lightly trafficked to have an impact.
6. Distance to Ångermanälven River*.* Because the mainstream of a river concentrates all flow within a catchment, it has an effect on slope stability, directly through fluvial erosion that undercuts riverbanks, and indirectly through tributaries, and surface runoff that creates fractures in soils for water to penetrate (Christiansson and Arnér 1993). A multi-buffer was used originating from the shores along the mainstream of the river as mapped by Lantmäteriet (2010) with a classification of 0–499 mm, 500–999 mm, and a subsequent 1 km interval.

Locations of 328 mass movements, mapped by the Swedish geotechnical institute in point format (SGI 2012) were placed above the six landscape parameters. The point data on mass movements was corrected and supplemented since a number of points lacked in accuracy regarding coordinates e.g., when points are located in the river. By using a DEM and a curvature-raster in combination, both with an original 2 × 2 m resolution, more accurate coordinates for these mass movements could be identified. Further scars from landslides and ravines were identified using the same layers and Google Earth as reference. In total, 495 landslide scars were used in the analysis.

For each parameter *p* a frequency ratio LSI_*p,k*_ representing the LSI for the classes *k* within every parameter is calculated as

1 $$ {\mathrm{LSI}}_{p, k}=\frac{N_{p, k}}{N}\frac{A}{A_{p, k}} $$

where *N*_*p*,*k*_ is the number of mass movements and *A*_*p*,*k*_ is the number of pixels for each class and parameter combination, and *N* and *A* are the total number of mass movements and total number of pixels within the study area, respectively. Equation (1) is based on previous studies by Lee and Talib (2005) and Shabanzadeh et al. (2011). Increasing values of LSI_*p*,*k*_ indicate an increased tendency for mass movements and hence increased landslide susceptibility, normalized by area and frequency of landslides, and applicable to the parameter and class considered. Hence the numerical values of LSI_*p*,*k*_ can be used to compare the relative influence of classes within each parameter, but do not allow for classes or parameters to influence each other. Note that classes covering very small areas and hence containing only a small number of pixels may result in high LSI_*p*,*k*_ values even for moderate number of mass movements; values for classes covering larger spatial distributions across the study area are therefore generally more reliable.

In order to include the stabilizing and destabilizing influence of classes between parameters and to create a slope instability map, weights *W*_*p* , *k*_ are calculated for every class as (Shabanzadeh et al. 2011)

2 $$ {W}_{p, k}=100\left(\frac{N_{p, k}}{N}-\frac{A_{p, k}}{A}\right) $$

The resulting weight values for every class are added together as an overlay showing the slope instability of every pixel within the study area in relation to other pixels. In contrast to LSI_*p*,*k*_ the *W*_*p* , *k*_ can take the form of both positive and negative values. The combined values are divided as discreet data into five classes where *W*_*p* , *k*_ ≤ 25 are classified as very low probability. The remaining four mass movement probability-classes were divided as: very low probability (<−25), low probability (−25 – −1), moderate probability (0–29), high probability (30–64), and very high probability (≥65).

### Validating the LSI

Results of the LSI is validated through comparisons of distribution between weight values for 125 randomly selected mass movement points (25%) chosen through research-randomizer (Urbaniak and Plous 2013) and which were not used to calculate the *W*_*p* , *k*_, the remaining 370 points used in the LSI, and 370 randomly created points. The distribution of weight values should be similar across the first two groups consisting of mass movement points, while the randomly created points should mirror the overall distribution of weight values across the map as a whole. Paired *T* tests between 3 groups will confirm that the difference in distribution is statistically significant.

### Overlay analysis

LSI-values were added to point-data of contaminated sites using “extract to points” in ArcGIS. Coordinates and metadata on 209 contaminated or potentially contaminated sites are available for within the study area. For each point, attribute data from SEPA (2002) was added for risk-class, branch-type, and branch-number. The branch information allows for further investigations on specific sites of interest by displaying the type of activity present at the location. The end result will be information on the type of contaminated sites that are exposed to slope instability, to what extent, and their location within the study area, in the form of maps and statistical data.

## Results

### Correlations between landscape parameters and mass movement occurrences along Ångermanälven river

The frequency ratio (LSI_*p*,*k*_) and weight values (*W*_*p* , *k*_) for all classes within the parameters are summarized in Table 1. For every class an *LSI*_*p*,*k*_-value >1 in combination with a positive *W*_*p* , *k*_-value indicates a positive correlation to mass movement occurrences within the study area. The results are based on a relative mass movement probability between the classes used in the study and are not an absolute measurement of the probability for mass movements to occur.

**Table 1:** Frequency ratio and weight values for parameters and classes

Slope gradient (degrees)	LSI_*p,k*_	*W*_*p*,*k*_	Curvature	LSI_*p*,*k*_	*W*_*p*,*k*_
0° to 5°	0.17	−54.51	≤−50*	40.26	1.32
6° to 10°	0.12	−12.38	−49 to −30	84.88	20.56
11° to 15°	1.59	4.52	−29 to −10	6.82	48.43
16° to 20°	4.37	10	−9 to 10	0.04	−80.2
21° to 25°	12.22	14.14	11 to 30	1.74	5.53
26° to 30°	18.72	10.74	31 to 50	9.95	1.95
31° to 35°	32.95	11	≥51*	136.93	3.49
36° to 40°	63.96	11.17			
41° to 45°^a^	192.54	4.53			
>45°^a^	19.99	0.04			
Land cover	LSI_*p*,*k*_	*W*_*p*,*k*_	Distance to roads	LSI_*p*,*k*_	*W*_*p*,*k*_
Mixed forest	4.72	27.71	≤100 m	1.91	3.62
Young forest	1.15	1.54	101 to 202 m	2.94	7.5
Broad-leaved forest	1.42	1.53	201 to 300 m	4.2	11.94
Coniferous forest	0.46	−22.2	301 to 400 m	5.31	15.57
Forest clearing	0.16	−9.92	401 to 500 m	2.94	6.79
Sparsely vegetated areas^a^	0	−0.003	>500 m	0.44	−45.43
Limnogenic wetlands^a^	0	−0.008			
Marshes	0.02	−11.01			
Arable land	3.57	8.36			
Pasture	3.89	4.82			
Fruit and berry plantations^a^	144.11	0.8			
Sand plains^a^	4.39	0.21			
Golf course^a^	0	−0.01			
Urban fabric	3.89	4.82			
Soil	LSI_*p*,*k*_	*W*_*p,k*_	Distance to river	LSI_*p,k*_	*W*_*p,k*_
Bedrock	0	−8.86	0 to 500 m	8	74.03
Felsenmeer and blocky coast	1.29	0.56	501 to 1000 m	1	0.1
Till	0.09	−44.91	1001 to 2000 m	0.1	−18.26
Gravel	2.44	2.79	2001 to 3000 m	0.13	−17.22
Sand	8.7	52.63	3001 to 4000 m	0	−19.52
Silt	2.91	9.93	>4000 m	0	−19.13
Clay	0.8	−0.37			
Peat	0.02	−12.08			
Filling^a^	5.11	−0.05			

LSI_*p*,*k*_ = frequency ratio, *W*_*p*,*k*_ = weight value

^a^Classes with few pixels/small spatial distribution in relation to the study area

The results indicate that mass movement probability can be tied mainly to five factors across all classes and parameters. These are a slope gradient ≥16°, a proximity to the river that is <500 m, a distance of <500 m from roads, concave surface curvature, and soils consisting of sand and silt. Areas with the lowest probability of mass movement are mainly located on ground with low slope gradient, plane surfaces, moraine soil, marshlands, and a distance of >1 km from the river. An LSI_*p*,*k*_-value of 0 indicates that no previous mass movements have been identified within the area covered by the class, and should therefore be considered as having an unknown susceptibility or no susceptibility to mass movement.

Across all parameters, 10 classes have a very small spatial distribution throughout the study area which has resulted in high frequency ratio-values when containing one or a small number of mass movement-points. This is because of normalization within the LSI_*p* , *k*_-equation between areal coverage and frequency of occurred mass movements. Weight values (*W*_*p* , *k*_) for these 10 classes are not impacted in the same way as the frequency ratio (LSI_*p* , *k*_) since a small spatial distribution results in lower weight values.

Results shown in Tables 2 and 3 indicate that 4.9% of the study area is exposed to a low or higher grade of mass movement probability. Forty-six (22%) of all contaminated sites are located within these exposed areas, of which a majority, 30 sites (14%) are situated on ground with a low or moderate risk. Three sites with a class 2 contamination risk (2nd highest) are located on ground with a very high probability of mass movement. These sites are identified as having the highest combined hazard when measuring both area instability and contamination.

**Table 2:** Distribution of mass movement probability-classes across the study area

Mass movement probability	Km^2^	Percentage
Very low probability	2324	95.09
Low probability	41	1.67
Moderate probability	36	1.47
High probability	20	0.81
Very high probability	23	0.94
	2444	99.98

**Table 3:** Contaminated areas exposed to slope instability

Mass movement probability	All contaminated areas		SEPA contamination risk-classification			
	Amount	%	Class 1	Class 2	Class 3	Class 4
Very low probability	163	78	3	63	68	29
Low probability	12	6	1	3	5	3
Moderate probability	18	8	0	6	6	6
High probability	8	4	0	4	2	2
Very high probability	8	4	0	3	3	2
Total	209	100	4	79	84	42

### Contaminated area instability

**Fig. 2:**
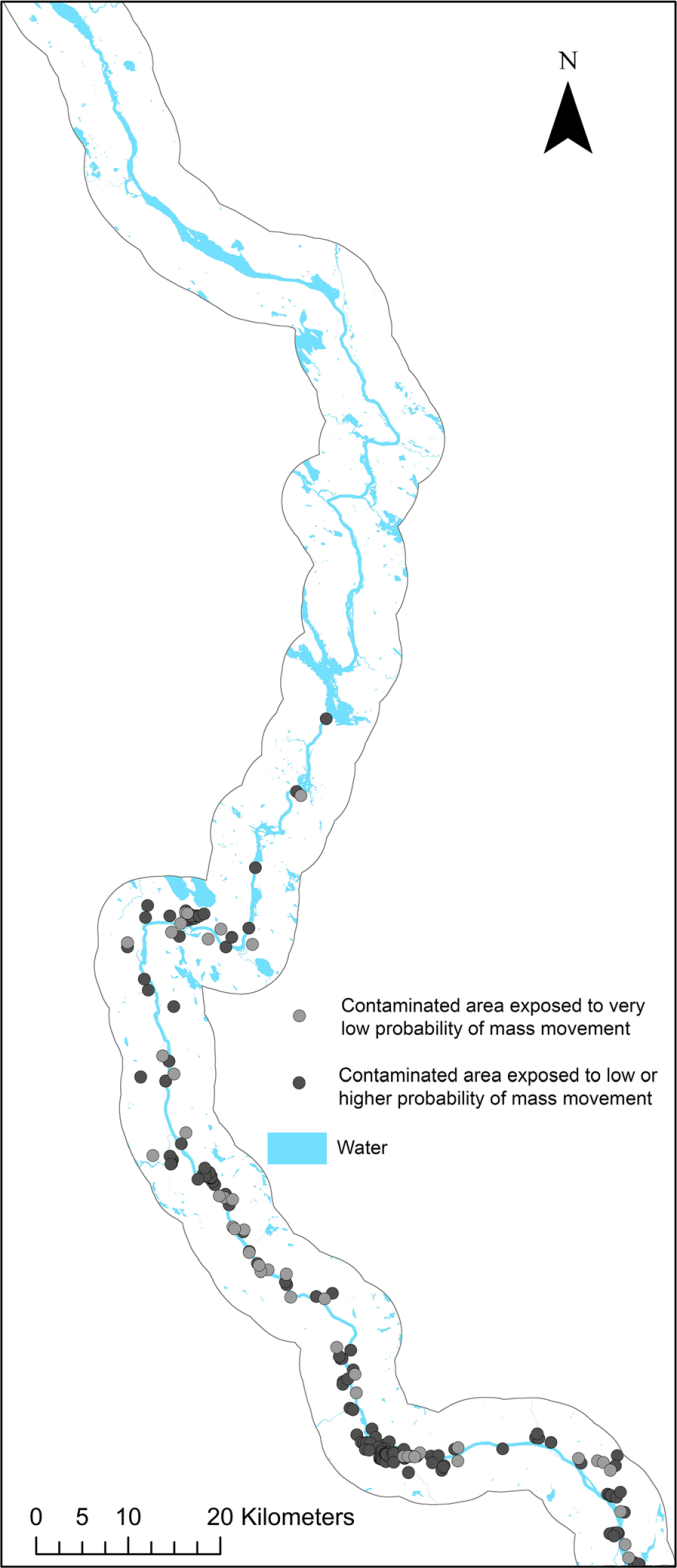
Contaminated sites within areas of low to very high probability of mass movement

**Fig. 3:**
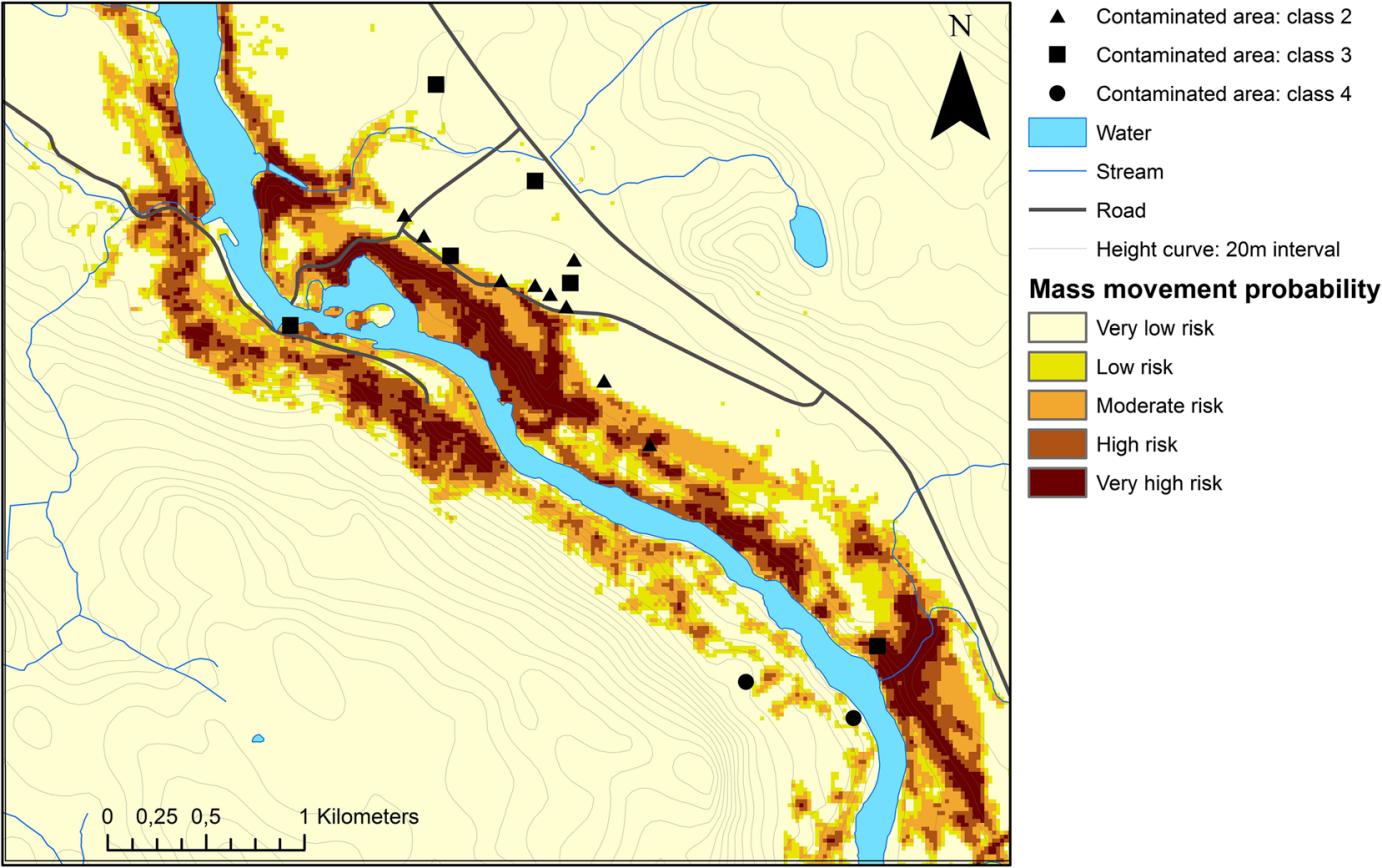
Local area close up projecting contaminated areas above mass movement probability

The matrix in Table 3 depicts the distribution of contaminated areas across different classes of slope instability. Sixteen contaminated sites, 8% of total, are located in areas with high and very high probability of mass movement occurrence, 30 sites or 14% of total are located in areas of moderate and low probability of mass movement occurrence, and 78% of the contaminated sites are located on ground with a very low probability for mass movements to occur. Figure 2 shows the spatial distribution of all contaminated sites across the study area classified as contaminated areas exposed to a very low probability, and contaminated areas exposed to ≥ low probability. The frequency of contaminated sites that overlap ground exposed to a low or higher probability of mass movement increases towards the south of the Ångermanälven River, markedly concentrated to beneath the highest coastline where the five main factors identified are more commonly occurring in combination. Figure 3 gives an example of a local area close up where detailed information on contamination risk classifications and mass movement probability are represented spatially. Every ground-pixel possesses a weight value and a *W*_*p* , *k*_ and a LSI_*p*,*k*_-value, and every point representing a contaminated area possesses information on risk-class, branch-type and branch-number, *W*_*p* , *k*_ and LSI_*p,k*_-values.

## Discussion

### The landslide susceptibility index

The results for the LSI show how mass movement probability along Ångermanälven River is determined mainly by five factors. Through an overlay with spatial data on contaminated sites the results further indicate that ground prone to mass movements is generally concentrated to areas adjacent to the southern stretches of the river, the same areas as a majority of contaminated and potentially contaminated sites.

The advantages and disadvantages of producing a bivariate LSI instead of using already available slope stability data should be considered in regards to the possible further use of the results, and the validity of the data sources used to produce it. The equations used could link individual classes to previous mass movements in isolation from each other, later combined as an overall weight value for each pixel. The method can therefore to a high degree account for circumstances where specific classes might need to interact with each other to produce instable ground conditions. Weight values for spatially overlapping classes can influence each other e.g., when a main factor for mass movement frequently overlaps with a less influential, or non-influential class to raise the weight value of the less influential one throughout the whole study area. Such possibilities will, if considered relevant, be discussed specifically for individual classes. The parameters used in the LSI are also non-dynamic over time in that they show the current state and spatial distribution of classes that can be susceptible to common triggering factors. Göransson et al. (2009) made use of available spatial data on landslide probability, which would generally produce faster results when mapping contaminated area instability. In such cases accuracy is determined by the acquired data on landslide probability in which individual parameters usually cannot be adapted or updated for improved accuracy. An LSI with site-specific parameters can in addition to the spatial distribution also describe the conditions and factors related to mass movements in different areas, as the data on parameters used to calculate mass movement probability is still available after the LSI has been produced. Data on mass movement probability can therefore be updated continuously and quickly, when newer or better-quality data on ground parameters or mass movements are made available, making it a dynamic and adaptive approach.

### Parameters and classes used in the landslide susceptibility index

A distance to the river of <500 m with a weight value (*W*_*p* , *k*_) of 74.03, and a frequency ratio (LSI_*p*,*k*_) of 8 was identified as having the strongest correlation to mass movement probability relative to all other classes and parameters. The class shows a clear negative relationship between the distance from the river and mass movement probability. Plausible influences on this relationship other than actual ground instability could be that the inventory of mass movements used in the LSI focused mainly on areas close to the river, or that scars along a riverbank were more easily identified when producing such an inventory. The high *W*_*p*,*k*_-value could also affect, and be affected by the fact that the other four classes also determined by the LSI-analysis as influential factors are to a high degree present in varying combinations within this 500 m proximity to the riverbanks. Fluvial processes such as riverbank erosion, accounted for in the distance to river-parameter might therefore have had the effect of adding to weight values of other classes such as strong slope gradient, strong curvatures, silt and sandy soils, all of which are often found overlapping within this 500 m proximity. This does not necessarily influence the results negatively as the slope instability is only represented as relative to other pixels in the study area. The effect would mainly serve to increase the range of values since negative weight values would have a similar influence. Furthermore, through normalizing when calculating weight values the results also show that the five most influential classes are statistically more frequently occurring in areas where most previous mass movements were identified. Pixels given a high or very high probability of mass movement could only be classified as such when most, or all of these classes were present within that area due to the threshold values set between classes of mass movement probability. This classification of mass movement probability also lowers the risk of ecological fallacies in the produced maps. Classes with strong negative weight values e.g., low curvature and flat surfaces cancel out high values caused by spatial generalization due to the use of discreet classes. This resulted in a case where flat and non-curved surfaces run a very small probability of being exposed to mass movements, even if they are located within e.g., <500 m proximity to the riverbeds. The factors identified by the LSI are in accordance with results of previous studies (Christiansson and Arnér 1993; Svensson et al. 2006; SGI 2014) of mass movements and their influencing factors along the river. Of the five main factors identified “distance to roads” is the only one that cannot be tied directly to earlier studies of mass movement within the region, and the effects of including this parameter have to be considered.

Within the distance to roads parameter all classes except >500 m show an even susceptibility to mass movement. The class >500 m could also have been affected by the factors mentioned above due to it covering a main part of the study area. Because of the steeper topography along the rivers southern stretches many roads run alongside riverbanks and could therefore have been affected by weight*-*values from the class <500 m distance to the river among others. This gives a higher degree of uncertainty regarding the distance to roads-parameter’s effect on slope stability, the risk being that most other influencing factors used in the LSI could affect it. Although some research (Srbulov 2010) points to that roads can effect ground instability, their weight values in this analysis are most likely affected by the road network’s location.

Mixed forest has not been determined as a main factor even though it has been given a moderately high weight value of 27.71. This is because of a high probability that this type of vegetation has shifted its spatial distribution since the CORINE land cover data was produced in 2002. However, the spatial distribution of mixed vegetation is mostly concentrated to areas along the river and gradually gives way to pine forest as the sand and clay soils are exchanged for moraine with an increased distance and relief away from the river.

### Location of the contaminated areas exposed to mass movement

Results shown in Tables 2 and 3 indicate that 22% of contaminated sites were located within the 4.9% of the study area that is exposed to a low or higher probability of mass movement. The most plausible reason for this is that the industrial infrastructure is concentrated mainly to areas adjacent to rivers in the southern part of the study area. The topography in the northwestern regions of the study area is significantly flatter along and further outwards from the riverbanks, which also allows for the few contaminated or potentially contaminated sites there to be located somewhat further from the river. Because the data on contaminated sites available for this study consisted of point data, the spatial extent of each site could not be considered in the analysis. Instead, the available point data indicate that at least part of a contaminated or potentially contaminated site is located on instable ground. If the spatial extent were to be included in the analysis, contaminated sites would cover larger areas, since point-coordinates do not have any spatial extent. Based on the produced results it is not possible to conclude whether the contaminated sites would cover more or less of the study areas stable or unstable ground if given their exact spatial extent. The results do however show that some part of each of the 46 overlapping contaminated sites are located on instable ground, to give a stable base for field verification.

A factor not accounted for in this study is the hydroelectric power plants and their distribution across the whole of Ångermanälven River. Such plants may function to divert pathways, or change the flow patterns in a river system, which in turn would affect the frequency, magnitude, and spatial distribution of mass movements along its river banks. Changes like this would also in many cases change the sediment transportation, i.e., the movement of sediment bound contaminants. It has been suggested that sediments, and thereby also contaminated sediments could reside in such river-systems for a longer period of time by hydroelectric power plants affecting the overall retention capacity of the river (Brydsten et al. 1990). A future need would therefore be the collection of data on the effects of hydroelectric plants and dams regarding water flow patterns and sediment transportation. Such data could prove useful in studies of contaminated area instability along the river itself, and as a means to make comparisons between the pathways and fate of contaminants in hydroelectrically exploited and unexploited rivers.

As was shown in studies by Göransson et al. (2009, 2012, 2014), mass movement along the industrially exploited Göta River has the potential of creating pathways for contaminants that has been mainly overlooked in Swedish environmental risk assessment. The results of this study indicate that there is a strong probability that the same risk exists along stretches of the Ångermanälven River as well. Göransson et al. (2009, 2012, 2014) studies of the Göta River focused mainly on its southern stretches, where landslides are known to be frequent. This study instead permits a relatively fast, larger regional overview, although at the cost of more detailed results. Industrial infrastructure and its exploitation of rivers in Sweden historically tend to expand when in closer proximity to cities and coasts. However, where watersheds do not share a similar geological or industrial history, i.e., the spatial distribution, frequency and magnitude of mass movements or contaminating activities differ; the same general assumption cannot be made. It should in any case be considered highly likely that many industrially exploited river basins around the world could be exposed to a varying degree of mass movement induced transport of contaminants into rivers. This would be reason for further developing a contaminated area instability-analysis, especially for use within watersheds with an industrial history, and which have a known presence of instable river banks. Examples of regions where the method presented in this paper could prove useful would be some of the industrially exploited regions within the Danube Basin, where contaminants (Literáthy et al. 2002; Kukucka et al. 2015), mass movements (Újvári et al. 2009; Mentes et al. 2009; Habersack et al. 2016), and hydroelectric plants are present in various combinations.

## Conclusions

This study shows that there is a strong probability that 8% of the 209 contaminated sites analyzed within the study area along the Ångermanälven River are at a high or very high probability of being exposed to slope processes. Results indicate that a disproportionate amount 46 (22%) of all contaminated sites are located within a 4.9% area of the study area, beneath the highest coastline, that is exposed to a low or higher probability of mass movements occurring. A landslide susceptibility index tied the mass movement probability mainly to five probability classes across all classes and parameters representing landscape features. These were a slope gradient ≥16°, a proximity to the river that is <500 m, a distance of <500 m from roads, concave surface curvature, and soils consisting of sand and silt. These results further imply a probability that other rivers along the Swedish east coast, sharing similar geological, physical, and industrial features, would be in need of similar assessments. A landslide susceptibility index proved a valuable complement to the overlay methods used by Göransson et al. (2009) by estimating slope instability through the use of spatial data on previous mass movements, a digital elevation model, a soil map, a land cover map, and a road map, in a case where slope instability data was not available. By providing such information from more generally available data, a first-step contaminated area instability-assessments become applicable also in regions that have not been studied and mapped for slope instability beforehand. This will help in further understanding the overall scale and distribution of contaminated area instability as a process, and thereby the scope of the problem as a whole.
